# Genome-Wide Identification of *N*6-Methyladenosine Associated SNPs as Potential Functional Variants for Type 1 Diabetes

**DOI:** 10.3389/fendo.2022.913345

**Published:** 2022-06-16

**Authors:** Yang Chen, Min Shen, Chen Ji, Yanqian Huang, Yun Shi, Li Ji, Yao Qin, Yong Gu, Qi Fu, Heng Chen, Kuanfeng Xu, Tao Yang

**Affiliations:** ^1^ Department of Endocrinology and Metabolism, The First Affiliated Hospital of Nanjing Medical University, Nanjing, China; ^2^ Department of Epidemiology and Biostatistics, School of Public Health, Nanjing Medical University, Nanjing, China; ^3^ Department of Emergency Medicine, The First Affiliated Hospital of Nanjing Medical University, Nanjing, China

**Keywords:** m6A (*N*6-methyladenosine), type 1 diabetes (T1D), single nucleotide polymorphism, autoimmune disease, intronic variant

## Abstract

**Objectives:**

N6-methyladenosine (m6A) is essential in the regulation of the immune system, but the role that its single nucleotide polymorphisms (SNPs) play in the pathogenesis of type 1 diabetes (T1D) remains unknown. This study demonstrated the association between genetic variants in m6A regulators and T1D risk based on a case-control study in a Chinese population.

**Methods:**

The tagging SNPs in m6A regulators were genotyped in 1005 autoantibody-positive patients with T1D and 1257 controls using the Illumina Human OmniZhongHua-8 platform. Islet-specific autoantibodies were examined by radioimmunoprecipitation in all the patients. The mixed-meal glucose tolerance test was performed on 355 newly diagnosed patients to evaluate their residual islet function. The functional annotations for the identified SNPs were performed in silico. Using 102 samples from a whole-genome expression microarray, key signaling pathways associated with m6A regulators in T1D were comprehendingly evaluated.

**Results:**

Under the additive model, we observed three tag SNPs in the noncoding region of the PRRC2A (rs2260051, rs3130623) and YTHDC2 (rs1862315) gene are associated with T1D risk. Although no association was found between these SNPs and islet function, patients carrying risk variants had a higher positive rate for ZnT8A, GADA, and IA-2A. Further analyses showed that rs2260051[T] was associated with increased expression of PRRC2A mRNA (*P* = 7.0E-13), and PRRC2A mRNA was significantly higher in peripheral blood mononuclear cell samples from patients with T1D compared to normal samples (*P* = 0.022). Enrichment analyses indicated that increased PRRC2A expression engages in the most significant hallmarks of cytokine-cytokine receptor interaction, cell adhesion and chemotaxis, and neurotransmitter regulation pathways. The potential role of increased PRRC2A in disrupting immune homeostasis is through the PI3K/AKT pathway and neuro-immune interactions.

**Conclusion:**

This study found intronic variants in PRRC2A and YTHDC2 associated with T1D risk in a Chinese Han population. PRRC2A rs2260051[T] may be implicated in unbalanced immune homeostasis by affecting the expression of PRRC2A mRNA. These findings enriched our understanding of m6A regulators and their intronic SNPs that underlie the pathogenesis of T1D.

## Introduction

Type 1 diabetes (T1D) is an autoimmune disease involving insulin-producing pancreatic islet beta cell prolonged destruction by autoreactive effector T lymphocytes (1). Although management with exogenous insulin changes T1D from an acutely fatal disease into a chronic disease, achieving glycemic control is still subject to significant limitations (2). The incidence of T1D continuously increases worldwide while varies a lot in different regions. In addition to environmental triggers, genetic factors likely play a vital role in the development of T1D. To date, nearly 60 risk loci have been identified in genome-wide association studies (GWAS), including MHC (major histocompatibility complex), INS, PTPN22 (protein tyrosine phosphatase N2), and so forth (3, 4). Most risk variants for T1D are located in noncoding regulatory regions (5) and are supposed to take part in altering gene expression. However, few studies have focused on potential functional variants that may affect N6-methyladenosine (m6A) methylation; the cellular mechanisms corresponding to their pathogenic processes are poorly understood.

N6-methyladenosine (m6A) is the most common internal modification of mRNA in eukaryotes. As a critical chemical mark, m6A regulates post-transcriptional gene expression by affecting RNA splicing, nuclear export, stability, and translation (6). The m6A RNA modification is reversible and can be dynamically modulated by a series of regulators. Among them, methyltransferase “writers”, m6A-binding proteins “readers”, and demethylase “erasers” are responsible for catalyzing, recognizing, and removing the m6A marks, respectively (7). Emerging studies have demonstrated that aberrant expression of m6A regulators such as METTL3, METTL14, and FTO can lead to dysregulated m6A modification, which is involved in the pathophysiology of various diseases, including cancers, immune disorders, viral infection, etc. (8–10). Given its importance in regulating gene expression and immune homeostasis, m6A modification could be involved in the pathogenesis of T1D.

Several single nucleotide polymorphisms (SNPs) located in m6A regulator genes have been identified as genetic variants related to different diseases, such as obesity (11), cancers (12), and autoimmune diseases (13). However, the association between genetic variants in m6A regulator genes and T1D risk remains elusive. To further investigate the potential pathogenesis of m6A modification in T1D, it is necessary to evaluate the functional variants in m6A modification.

Therefore, we conducted a case-control study of 1005 patients with T1D and 1257 healthy controls in a Chinese population to evaluate the association between tagging SNPs in m6A regulators and T1D risk. Next, we investigated the association between risk loci and clinical features. To verify whether these m6A regulator SNPs have potential regulatory effects on gene expression and whether these disturbances may lead to T1D, we performed eQTL and differential expression analyses using the GTEx portal and the GEO public dataset.

## Materials and Methods

### Study Subjects

A total of 1005 patients with T1D and 1257 healthy controls were recruited from the First Affiliated Hospital of Nanjing Medical University between January 2008 and December 2016. All patients with T1D had been diagnosed according to the American Diabetes Association criteria and had a positive test for at least one pancreatic islet-specific autoantibody (ZnT8A, GADA, IA-2A, IAA) (14). Those with the clinical features of latent autoimmune diabetes in adults were excluded. The inclusion criteria for the non-diabetic healthy controls have been described previously (15). Briefly, the control subjects (422 female, 835 male; mean age 41.96 ± 16.36 years) were enrolled from healthy individuals in Jiangsu province without the medical history of diabetes or evident autoimmune diseases. Written informed consent was obtained from all participants or their guardians. Each participant was interviewed to collect demographic information and peripheral blood samples. All subjects were unrelated members of a Chinese Han population. Their clinical characteristics are shown in **Supplementary Table 1**. This study was approved by the Ethics Committee of the First Affiliated Hospital of Nanjing Medical University.

### Selection of m6A Regulator Tag SNPs and Genotyping

The m6A RNA methylation regulators consist of 10 writers (METTL3/14/16, WTAP, VIRMA, ZC3H13, CBLL1, RBM15/15B, ZCCHC4), two erasers (FTO, ALKBH5), and 16 readers (YTHDC1/2, YTHDF1/2/3, HNRNPC/G, HNRNPA2B1, IGF2BP1/2/3, FMR1, PRRC2A, LRPPRC, ELAVL1, ZFP217) (6, 9, 16). A total of 21,912 SNPs in the m6A regulator gene and within ±10 kb flanking regions were retrieved from the 1000 Genomes project in the Chinese Han population (CHB) data (https://www.internationalgenome.org/). Genomic DNA extracted from peripheral blood lymphocytes was genotyped using the Illumina Human OmniZhongHua-8 platform, providing 787,224 qualified SNPs. Then we extracted m6A regulator variants from it based on quality control and imputation protocols (3). First, SNPs with minor allele frequency < 0.05 or a Hardy-Weinberg equilibrium (HWE) < 0.05 were excluded. Next, Haploview 4.2 software was used to select tag SNPs with an r^2^ threshold > 0.4. Finally, 39 SNPs with call rates > 95% were selected. Detailed information on these SNPs is presented in **Supplementary Table 2**. In addition, variants were excluded from subsequent association analyses with differences in effect size compared to the European ancestry meta-analyzed UCSD T1D-GWAS (5).

### Pancreatic Islet-Specific Antibodies Detection

Autoantibodies to ZnT8, GAD65, and IA-2 were detected separately using protein A radio-binding assays (1). Serum IAA was only tested within two weeks after diagnosis using an ELISA kit (Biomerica, USA) to avoid interference of injected insulin.

### Mixed-Meal Tolerance Test

A total of 355 eligible newly diagnosed T1D patients underwent a mixed-meal tolerance test (MMTT) to assess their residual β cell function. Plasma C-peptide concentrations at baseline, 60 min, and 120 min after an MMTT were measured using an automated chemiluminescence assay (Roche, Switzerland). The 2-hour C-peptide area under the curve (AUC) was calculated with the trapezoidal method.

### Functional Annotation for m6A Regulator Loci

The GTEx v8 Portal (http://www.gtexportal.org/home/) was searched to conduct the expression quantitative trait loci (eQTL) analyses. Then the functional annotations for the identified SNPs were performed with HaploReg v4.1 (https://pubs.broadinstitute.org/mammals/haploreg/haploreg.php). Expression profiles were downloaded from the GEO database [GSE9006 (17) and GSE72492 (18)]. We compared the expression data of PRRC2A and YTHDC2 in peripheral blood mononuclear cell (PBMC) samples between 79 T1D and 23 healthy controls. Additionally, the YTHDC2 mRNA expression in pancreatic samples was compared between 10 T1D and 7 controls. Then gene expression data of PBMC samples from patients with T1D were divided into high- and low- groups according to the median PRRC2A expression level. The differentially expressed genes (DEGs) between high and low PRRC2A expression groups were identified using the limma package (19), and the thresholds were |log2FoldChange| > 0.5 and *P* value < 0.05. GO and KEGG pathway analysis was performed on the DEGs between high and low PRRC2A expression groups with the clusterProfiler R package (20). A false discovery rate (FDR) cutoff of 0.1 was used for statistical significance. To elucidate the differences between high- and low- PRRC2A groups, gene set enrichment analysis (GSEA) was performed using the molecular signature database (MSigDB) collections: c2.all.v7.2.symbols, c7.all.v7.2.symbols by R package clusterProfiler (20, 21). Normalized enrichment scores (|NES| > 1), adjusted *P*-values < 0.05 and FDR q value < 0.25 were considered statistically significant.

### Statistical Analysis

Departures from the HWE in control subjects were verified by the goodness-of-fit χ2 test. OR (95% CI) and *P* values were derived from logistic regression analyses with adjustment for sex and the ten principal components under the assumption of an additive genetic model. The principal components are described in a previous report (3). Multivariate logistic regression analyses for islet function were also performed to assess its effects on T1D susceptibility. The *P* values of eQTL were obtained from the linear regression model. Differences in the distribution of categorical variables were evaluated with the χ2 test, and the continuous variables were compared with Welch’s t-test. In cases of more than two groups, one-way ANOVA or the Kruskal-Wallis test was used. All analyses were performed using R 4.0.5 and plink1.07. Two-tailed *P* values below 0.05 were considered statistically significant.

## Results

### SNPs in PRRC2A and YTHDC2 Genes Are Associated With T1D Risk

Among all the 39 selected SNPs, three tag SNPs, namely, rs2260051 (A>T), rs3130623 (C>T), and rs1862315 (A>C) were significantly associated with T1D risk under the addictive model (*P* = 7.87E-05 for rs2260051, *P* = 3.72E-13 for rs3130623, and *P* = 0.045 for rs1862315). The results were further validated in an independent UCSD T1D-GWAS (5) dataset containing 73,011 individuals. Among these, rs2260051 and rs3130623 reached the threshold of GWAS significance (**Table 1**). In addition, we calculated the cumulative risk score across the three SNPs. As shown in **Table 2**, individuals who carry 3-4 risk alleles are associated with higher incidences of T1D (OR = 1.83, 95% confidence interval (CI): 1.52-2.21, *P* = 1.98E-10). A stronger trend was found for individuals with 5 or 6 risk alleles (OR = 4.92, 95% CI: 2.07-13.03, *P* = 5.65E-04). It suggests a significant association between cumulative risk score in PRRC2A and YTHDC2 gene with T1D risk in a Chinese Han population (*P*_trend_ = 1.93E-12).

**Table 1:** Associations between the m6A regulator SNPs in PRRC2A, YTHDC2 gene, and T1D risk.

SNPs	Position(hg19)	Allele	EAF		Genotype distribution^a^		Additive model		UCSD T1D-GWAS^c^	
			T1D	Controls	T1D	Controls	OR(95%CI)^b^	*P* value	OR	*P* value
rs2260051	31591918	A/T	0.71	0.66	511/405/89	548/569/140	1.31 (1.15,1.50)	7.87E-05	1.49	8.75E-147
rs3130623	31597700	C/T	0.21	0.12	39/336/630	18/266/973	1.90 (1.60,2.26)	3.72E-13	2.01	7.49E-282
rs1862315	112849801	A/C	0.22	0.20	39/356/610	40/411/806	1.17 (1.00,1.37)	4.56E-02	1.05	6.70E-03

a Individuals homozygous for the effect allele/heterozygous/homozygous for the reference allele.

b OR (95% CI) and P values were derived from logistic regression analyses with adjustment for sex and the ten principal components under the assumption of an additive genetic model.

c The results were further validated in an independent UCSD T1D-GWAS (5) dataset containing 73,011 individuals.

**Table 2:** Associations between the m6A regulator SNPs cumulative risk score and T1D risk in a Chinese population.

Genetic risk score	No. cases/Total	OR(95%CI)^a^	*P* value
0-2	571/1469	Ref	
3-4	415/767	1.83(1.52-2.21)	1.98E-10
5-6	19/26	4.92(2.07-13.03)	5.65E-04
*P* value for trend			1.93E-12

a OR (95% CI) and P values were derived from logistic regression analysis with adjustment for sex and the ten principal components under the assumption of an additive genetic model.

### The Significant Association Between m6A Regulator SNPs and Islet Autoimmunity

To explore the potential effects induced by m6A regulator SNPs, we also evaluated the association between the genotype distribution of the three SNPs and the positivity of islet-specific autoantibodies in 1005 T1D patients. As shown in **Table 3**, after adjusting for sex and the ten principal components, ZnT8A, GADA, and IA-2A were all positively associated with rs2260051 and rs3130623. Patients with T1D who carried a high cumulative risk score had a higher frequency of islet autoantibody positivity. However, there was no significant association between YTHDC2 rs1862315 and islet autoantibody positivity (all *P* > 0.05).

**Table 3:** Associations of the m6A regulator SNPs with islet autoantibody positivity.

Group	rs2260051			rs3130623			rs1862315			Genetic risk score		
	OR(95%CI)^a^	*P* value	*P* ^b^	OR(95%CI)^a^	*P* value	*P* ^b^	OR(95%CI)^a^	*P* value	*P* ^b^	OR(95%CI)^a^	*P* value	*P* ^b^
**Gender**			0.386			0.329			0.255			0.908
** Male**	1.25 (1.05-1.49)	0.012		1.81 (1.45-2.25)	1.40E-07		1.27 (1.04-1.56)	0.020		1.34 (1.20-1.50)	1.41E-07	
** Female**	1.42 (1.12-1.81)	0.004		2.18 (1.61-2.97)	5.24E-07		1.05 (0.80-1.37)	0.739		1.35 (1.18-1.56)	2.10E-05	
**Antibody positivity**			0.968			0.671			0.789			0.702
** ZnT8A**	1.28 (1.05-1.56)	0.013		1.71 (1.33-2.19)	2.58E-05		0.98 (0.78-1.23)	0.881		1.24 (1.10-1.40)	0.001	
** GADA**	1.32 (1.11-1.58)	0.002		1.97 (1.59-2.44)	7.55E-10		1.07 (0.87-1.31)	0.520		1.32 (1.19-1.47)	2.81E-07	
** IA-2A**	1.29 (1.06-1.57)	0.013		1.95 (1.52-2.49)	1.12E-07		1.09 (0.87-1.38)	0.444		1.32( 1.16-1.49)	1.11E-05	
** Ab=1**	Ref			Ref			Ref			Ref		
** Ab>1**	0.94 (0.77-1.15)	0.541		0.92 (0.74-1.16)	0.485		0.89 (0.71-1.11)	0.294		0.93 (0.83-1.04)	0.225	

a OR(95% CI) and P values were derived from logistic regression analysis with adjustment for sex and the top ten principal components of ancestry under the assumption of an additive genetic model.

b P for heterogeneity test between subgroups.

### No Significant Correlation Between m6A Regulator SNPs and Residual C-Peptide

To assess the association between m6A regulator SNPs and residual islet function, we conducted an MMTT on 355 newly diagnosed patients with T1D. Generalized linear model analyses were performed to determine statistical significance and beta coefficients for age at onset, disease duration, body mass index, positivity of islet-specific autoantibodies, and residual islet β-cell function (fasting C-peptide, random C-peptide, or area under the C-peptide curve). As is shown in **Table 4**, there were no significant associations between m6A regulator SNPs and residual islet β-cell function (*P* > 0.05).

**Table 4:** Associations of the m6A regulator SNPs with residual islet function in newly diagnosed patients with T1D.

Traits	rs2260051			rs3130623			rs1862315			Genetic risk score			
	β^a^	SE	*P* value	β^a^	SE	*P* value	β^a^	SE	*P* value	β^a^	SE	*P* value
**Age at diagnosis (yrs.)**	-0.556	1.097	0.613	-2.517	1.178	0.033	1.074	1.185	0.366	-0.558	0.611	0.362
**BMI (kg/m2)**	-0.150	0.397	0.706	-0.364	0.445	0.414	0.343	0.429	0.424	-0.048	0.228	0.833
**Duration (yrs.)**	-3.60E-05	7.12E-05	0.613	-4.05E-05	7.74E-05	0.601	5.60E-05	7.75E-05	0.471	-7.21E-06	3.98E-05	0.856
**Number of Autoantibody positivity**	-0.016	0.094	0.866	-0.079	0.104	0.450	-0.231	0.104	0.027	-0.086	0.053	0.107
** Fasting C-Peptide (pmol/L)**	28.840	17.110	0.093	-15.124	18.990	0.427	5.840	18.798	0.756	6.713	9.632	0.487
** Random C-peptide (pmol/L)**	31.000	79.190	0.696	-154.290	81.720	0.061	55.880	81.080	0.492	-17.470	43.960	0.692
C-Peptide AUC (pmol/L*h)	59.600	92.380	0.520	-147.680	97.460	0.132	31.940	94.640	0.736	-13.680	53.410	0.798

a Results were reported as standardized β coefficient. SE, standard error. Asterisk (*) indicates multiplication sign.

### Rs2260051 Was Associated With Increased Expression of PRRC2A

**Figure 1:**
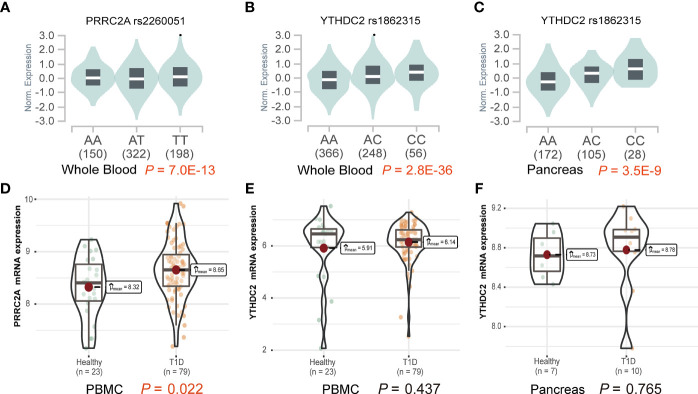
Effects of variants on gene expression of PRRC2A and YTHDC2. **(A)** The effect allele of rs2260051 was significantly associated with increased expression of PRRC2A in whole blood samples. The minor allele of rs1862315 was significantly associated with increased expression of YTHDC2 in **(B)** whole blood and **(C)** pancreas. **(D)** The expression levels of PRRC2A mRNA were significantly higher in the peripheral blood mononuclear cell (PBMC) samples from type 1 diabetes (T1D) than the healthy control. No significant differences in YTHDC2 between T1D and healthy control from **(E)** PBMC or **(F)** pancreatic samples.

Functional explorations using HaploReg annotated these SNPs to evaluate how they confer disease susceptibility. Rs1862315 acted as a promoter for H3K4me3, H3K9ac, and H3K27ac in different immune cell subsets, including T cell, B cell, monocyte, etc. The other two SNPs, rs2260051 and rs3130623, are also located in the enhancer histone marks for H3K4me1 and H3K27ac. (**Supplementary Table 3**) We also found that the risk allele of rs2260051 was associated with increased expression of PRRC2A in the whole blood sample (*P* = 7.0E-13) (**Figure 1A**), while rs1862315 was associated with increased expression of YTHDC2 both in whole blood (*P* = 2.8E-36) (**Figure 1B**) and pancreas (*P* = 3.5E-9) (**Figure 1C**). Moreover, using transcriptome dataset from the GEO, we found that the mRNA expression of PRRC2A was increased in PBMC samples from patients with T1D relative to the controls (*P* = 0.022) (**Figure 1D**). However, there was no significant difference in YTHDC2 expression between T1D and healthy, neither from the PBMC samples (**Figure 1E**) nor the pancreatic samples (**Figure 1F**). These results provide evidence that the three m6A regulator SNPs are in the functional promoter or enhancer region of the PRRC2A and YTHDC2 genes. Furthermore, rs2260051 may induce T1D by regulating the expression of PRRC2A.

### Increased Expression of PRRC2A Associated With Disturbed Immune Homeostasis

**Figure 2:**
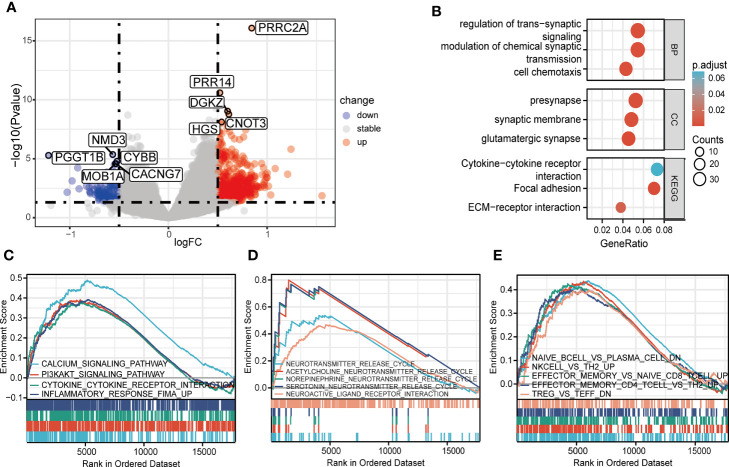
The identification and functional enrichment of differentially expressed genes based on PRRC2A mRNA expression in type 1 diabetes. **(A)** Volcano plot of the differentially expressed genes (DEGs) between groups exhibiting high and low levels of PRRC2A mRNA. **(B)** Significantly enriched Gene Ontology (GO) and Kyoto Encyclopedia of Genes and Genomes (KEGG) pathway terms of PRRC2A associated DEGs. **(C)** Gene set enrichment analysis (GSEA) indicated that high expression level of PRRC2A was correlated with calcium signaling, PI3K/AKT signaling, cytokine-cytokine receptor interaction, and inflammatory response pathways. **(D)** GSEA indicated that high expression level of PRRC2A was correlated with neuroactive ligand-receptor interaction, and neurotransmitter (acetylcholine, norepinephrine, serotonin) release cycle pathways. **(E)** GSEA indicated that high expression level of PRRC2A was correlated with downregulation of Treg and Th2, and upregulation of effector memory CD4+ T, CD8+ T cells, NK cells, and plasma cells.

A total of 800 DEGs (668 upregulated and 132 downregulated, **Supplementary Table 4**) were identified between the high and low PRRC2A expression groups (**Figure 2A**). To predict the functions of the interactive genes of PRRC2A, Gene Ontology (GO) and Kyoto Encyclopedia of Genes and Genomes (KEGG) analyses were also performed. We found that cytokine-cytokine receptor interaction involved in immune response, cell chemotaxis pathways involved in cell adhesion, and trans-synaptic signaling involved in neurotransmitter regulation were significantly enriched by PRRC2A-associated DEGs (**Figure 2B**). Moreover, gene set enrichment analysis (GSEA) between high and low PRRC2A expression groups revealed significant differences (FDR < 0.25, *P* < 0.05, and |NES| >1) in the enrichment of MsigDB collection. **Figure 2C** and **Supplementary Table 5** show that calcium signaling, PI3K/AKT signaling, cytokine-cytokine receptor interaction, and inflammatory response pathways were significantly enriched in T1D with high PRRC2A expression. In addition, neuroactive ligand-receptor interaction, and neurotransmitter release cycle pathways (acetylcholine, norepinephrine, serotonin) were significantly enriched in T1D with high PRRC2A expression (**Figure 2D** and **Supplementary Table 5**). Downregulation of Treg and Th2 were also enriched in the PRRC2A high-expression group. In contrast, upregulation of effector memory CD4+ T, CD8+ T cells, NK cells, and plasma cells were differentially enriched in PRRC2A high-expression phenotypes (**Figure 2E** and **Supplementary Table 5**).

## Discussion

Previous studies have established the important role of m6A modification in modulating gene expression that regulates T cell differentiation, proliferation, and cytokine production (22, 23). Taking the importance of m6A regulators in shaping balanced immune homeostasis into account, allelic variants in m6A regulators could be genetic risk factors for autoimmune disease. Polymorphisms in the ALKBH5 (24) and METTL21B (25) genes have been identified as genetic variants associated with autoimmune thyroid disease and multiple sclerosis. However, little is found in the literature on m6A regulator gene susceptibility in T1D. In this study, we genotyped 21,912 SNPs in 28 m6A regulator genes and evaluated their association with T1D risk in a case-control study. We found that three SNPs within the intronic region of PRRC2A and YTHDC2 were significantly associated with a higher risk for T1D.

The proline-rich coiled-coil 2A (PRRC2A) gene, also known as BAT2 (26), is located in the human major histocompatibility complex (MHC) class III region. It has been newly recognized as an m6A reader in controlling neural development *via* regulating the stability of the Olig2 mRNA (27). Several reports have shown that microsatellite repeats and missense polymorphisms in the PRRC2A gene conferred increased risk in various immune-related diseases, including type 1 diabetes, rheumatoid arthritis, coeliac disease, lupus nephritis (28–31). Masao et al. first reported that BAT2 microsatellite alleles are associated with the age-at-onset of insulin-dependent diabetes mellitus (28). The type 1 Diabetes Genetics Consortium (T1DGC) study also detected seven missense SNPs in the BAT2 gene associated with type 1 diabetes (32). This study further found two independent intronic variants in the PRRC2A gene, rs2260051, and rs3130623, associated with T1D risk. Both loci were identified in the Chinese population and validated in an independent European ancestry UCSD T1D-GWAS dataset (5). Over 95% of the variants in high LD were in the intronic region, making it difficult to elucidate their functional roles in the pathogenesis of human disease (33). Based on eQTL analysis, we found that the rs2260051 may influence the mRNA expression level of PRRC2A. Interestingly, rs3130623 is in moderate linkage disequilibrium with another PRRC2A variant, rs2736157, which was recently reported to be associated with neuromyelitis optica and multiple sclerosis in a Chinses cohort (34). Thus, these PRRC2A variants may provide insight into the pathogenesis of dysregulated m6A modification in autoimmune diseases.

As an N6-methyladenosine reader, YTHDC2 can selectively bind RNA with m6A modification. It plays a significant role in enhancing the translation efficiency of its targets and decreasing their mRNA abundance (35). Several studies have reported the critical functions of YTHDC2 during spermatogenesis and oncogenesis (12, 36). Daniele et al. also reported YTHDC2 gene could be involved in pancreatic cancer susceptibility (37). In this study, we identified rs1862315, in the promotor of YTHDC2, was significantly associated with the susceptibility of T1D. Further functional annotation in silico demonstrated that rs1862315 upregulates YTHDC2 in the whole blood and pancreas sample. However, no significant difference was found in YTHDC2 expression between T1D and healthy controls.

We also showed that a genetic score based on the cumulative effects of variants is associated with increased risk for T1D. It has been demonstrated that T1D is a polygenic disease, and the combination of multiple SNPs may reveal a greater risk than single SNP (38). In addition, multiple variants of a single gene locus can have an additive effect on affecting gene expression (39). We and others previously found that a higher genetic risk score (GRS) was associated with lower fasting C-peptide levels at diagnosis (3). It also resulted in a sharper decline in residual β-cell function following the T1D diagnosis (40). However, this study did not observe any association between risk alleles in PRRC2A or YTHDC2 and lower residual islet function. This might indicate that other SNPs involved in the GRS model but not PRRC2A or YTHDC2 risk alleles affect β-cell function.

Islet autoantibodies are important markers of islet autoimmunity, indicating disease progression and diagnosis. However, there is considerable interindividual variability in the progression rate of islet beta cell destruction (41). Genetic susceptibility is likely to play a significant role in it. Several studies have identified correlations between immune-related variants and the positive rate of islet autoantibodies, such as IL27-IA2A, IFIH1-IA2A, IL2RA-GADA, IL1B-ZnT8A, and CTLA4-IA2A (15, 42, 43). Our results confirm an association between PRRC2A risk alleles and islet autoantibody positivity. It indicates that PRRC2A risk variants might regulate autoimmunity and have the potential to be biomarkers of T1D progression.

In our findings, the risk variant of rs2260051 was significantly associated with enhanced expression of PRRC2A. We conduct bioinformatic analyses to investigate the function of PRRC2A in T1D using PBMC samples from new-onset patients with T1D. On the one hand, KEGG and GSEA analysis found cytokine-cytokine receptor interaction and PI3K/AKT signaling pathway were significantly enriched in T1D with high PRRC2A expression. Prior studies have noted the importance of the PI3K/AKT signaling pathway in regulating immunity. During the adaptive immune response, TCR or BCR stimulation activates the PI3K/AKT pathway, which takes part in the regulation of lymphocyte differentiation in turn (44). The GSEA results also suggested that higher expression of PRRC2A may enhance the number or function of pathogenic immune cells (effector memory T cells, NK cells, and plasma cells) while limiting the regulatory T cells. These findings suggested that increased PRRC2A in T1D was positively correlated with immune response and lymphocyte differentiation. On the other hand, neurotransmitter regulation was enriched by PRRC2A-associated DEGs. Recently studies revealed the role of neurotransmitters in the modulation of the immune system. Immune cells can synthesize and release neurotransmitters such as acetylcholine and catecholamines. The receptors of these neuromodulators have also been identified on monocytes, lymphocytes, etc. In addition, cytokine receptors are also expressed on sensory neurons (45). These findings are in accord with the present study, which indicates that increased expression of PRRC2A may be involved in imbalanced immune homeostasis in T1D through the PI3K/AKT pathway and neuro-immune interactions.

Our study was the first to report an association between the variants in m6A regulator genes and T1D risk. These results should be interpreted with caution. Although rs2260051 and rs3130623 in the PRRC2A gene reached the threshold of GWAS significance in an independent validation, the significance of YTHDC2 rs1862315 was uncorrected for multiple comparisons. Although we elaborated the functional annotations of the m6A regulator SNPs based on the GTEx Portal and RNA expression profiling from the GEO database, they could not be directly validated due to the absence of experiments. Further studies are needed to clarify the biological mechanisms.

Overall, this study found that m6A regulator SNPs in the functional elements of PRRC2A and YTHDC2 gene, including rs2260051, rs3130623, and rs1862315, were associated with increased risk for T1D. Of these variants, rs2260051 and rs3130623 showed significant association with islet autoantibody positivity in T1D individuals. For rs2260051, the risk allele was associated with increased expression of PRRC2A. Transcriptomic analyses confirmed that higher PRRC2A expression correlates with disturbed immune homeostasis. These findings might inspire further research to investigate the roles of PRRC2A and m6A regulator SNPs in the development of T1D.

## Data Availability Statement

The genotype data presented in the study are deposited in the GWAS Catalog repository, accession number GCST008377, GCST90014023. The RNA microarray data presented in the study are deposited in the GEO repository, accession number GSE9006, GSE72492. Further inquiries can be directed to the corresponding author.

## Ethics Statement

The studies involving human participants were reviewed and approved by Ethics Committee of the First Affiliated Hospital of Nanjing Medical University. Written informed consent to participate in this study was provided by the participants’ legal guardian/next of kin.

## Author Contributions

YC performed data analysis and drafted the initial manuscript. MS validated the results and helped with the data visualization. CJ and YH performed data analysis. YS, LJ, and YQ helped prepare for data collection and the literature search. YG and QF helped with the manuscript review and editing. HC contributed to the laboratory testing. KX worked on the methodology and supervision. TY designed the study and is the guarantor of this work. As such, TY has full access to all the study data and takes responsibility for the integrity of the data and the accuracy of the data analysis. All authors contributed to the article and approved the submitted version.

## Funding

This study was supported by the National Natural Science Foundation of China [Grant number: 81900708, 81830023, 81530026, 82000747], the National Key Research and Development Program of China [Grant number:2016YFC1305004], and the Postgraduate Research & Practice Innovation Program of Jiangsu Province [Grant number: JX10213850]. The sponsors had no role in the study design; the collection, analysis, and interpretation of data; the writing of the report; or the decision to submit the article for publication.

## Conflict of Interest

The authors declare that the research was conducted in the absence of any commercial or financial relationships that could be construed as a potential conflict of interest.

## Publisher’s Note

All claims expressed in this article are solely those of the authors and do not necessarily represent those of their affiliated organizations, or those of the publisher, the editors and the reviewers. Any product that may be evaluated in this article, or claim that may be made by its manufacturer, is not guaranteed or endorsed by the publisher.
